# Intimate partner sexual violence and early resumption of sexual intercourse among married postpartum women in Ethiopia: a survival analysis using Performance Monitoring for Action data

**DOI:** 10.3389/fgwh.2025.1499316

**Published:** 2025-04-30

**Authors:** Eyob Tilahun Abeje, Fekade Demeke Bayou, Fekadeselassie Belege Getaneh, Lakew Asmare, Abel Endawkie, Alemu Gedefie, Amare Muche, Anissa Mohammed, Aznamariam Ayres, Dagnachew Melak

**Affiliations:** ^1^ Department of Epidemiology and Biostatistics, School of Public Health, College of Health Sciences, Wollo University, Dessie, Ethiopia; ^2^ Department of Pediatrics and Child Health Nursing, College of Medicine and Health Sciences, Wollo University, Dessie, Ethiopia; ^3^ Department of Medical Laboratory Science, College of Medicine and Health Sciences, Wollo University, Dessie, Ethiopia

**Keywords:** early resumption of sexual intercourse, time to resume sex, intimate partner violence, pressured sex, forced sex, postpartum women, survival analysis

## Abstract

**Introduction:**

Many women worldwide resume sexual intercourse soon after childbirth, often before the recommended six-week recovery period. Early postpartum intercourse poses health risks, including infections and delayed healing. This study aims to assess the timing of resuming sexual intercourse and its predictors among postpartum women in Ethiopia using PMA data.

**Methods:**

The data was from the Performance Monitoring for Action (PMA) project, a cross-sectional design followed by cohort follow-up, employed to analyze the sociodemographic and reproductive characteristics of women aged 15–49. Pregnant women and those up to nine weeks postpartum at baseline were included in the study. Descriptive statistics and Cox proportional hazard model were used for analysis using R 4.4.1 software. Proportional hazard assumption was assessed using graphical and statistical tests. The model fitness was checked using martingale residual plot.

**Results:**

The study found that 29% of participants resumed sexual intercourse before the recommended 42 days postpartum, while 91% resumed by 68 days. The median survival time was 8 weeks (57 days). The hazard of early sexual resumption was 5.56 times higher among women who experienced intimate partner violence compared to those who did not.

**Discussion:**

Early sexual resumption among postpartum women in Ethiopia was high. Intimate Partner violence was a significant predictor of early sexual resumption. It is better to promote IPV prevention and postpartum couple counseling to support safe and consensual sexual resumption.

## Introduction

Giving birth leads to various transformations in a mother's physical condition and overall state, such as fatigue, mood swings, and alterations in sexual function. The time after giving birth, known as the postpartum period, is crucial for a mother's body to recover and undergo physical and emotional adjustments. Although there is variation in reports regarding women's sexual intercourse post-delivery, studies suggest that sexual function tends to normalize between 5 and 8 weeks following childbirth (1–3).

Globally, many women resume sexual intercourse soon after childbirth, often before the recommended six-week recovery period. Although many guidelines recommend a waiting period for physical healing before resuming sexual intercourse, a significant number of women engage in sexual relations sooner than recommended (4–10). The prompt exploration of sexual intercourse following childbirth is crucial yet frequently neglected in postpartum health, especially in Ethiopia. Recent research shows that a notable proportion of women after childbirth participate in sexual intercourse in the initial six weeks post-delivery, with figures varying from 20% up to 70% (4, 11, 12).

Engaging in sexual intercourse shortly after giving birth can cause serious health problems for women, such as both physical and psychological complications. These risks include infections, prolonged physical healing, and particular sexual health issues such as dyspareunia and vaginal dryness, leading to intimacy-related discomfort and anxiety (1, 13, 14). During the postpartum phase, it is important to allow for healing from the physical effects of pregnancy and childbirth, as engaging in sexual intercourse early can hinder this recovery process. Moreover, early engagement in sexual intercourse can lead to heightened stress, reduced quality of life, and tension in relationships, which can exacerbate the long-term health risks for both the female and her partner (12, 15–17).

Previous studies have identified factors influencing the timing of postpartum sexual intercourse, encompassing cultural, societal, and economic conditions, as well as the partner's influence and access to healthcare services. Women who receive inadequate guidance during postpartum care or lack family support are more likely to resume sexual intercourse prematurely. Additionally, maternal education, delivery type, parity, and breastfeeding practices significantly affect postpartum sexual timing, with higher education levels associated with delayed resumption (17–20).

Health organizations, including the World Health Organization (WHO), suggest refraining from sexual intercourse for a minimum of four to six weeks after giving birth to promote proper healing, highlighting the importance of evaluating a woman's physical and emotional preparedness for intimacy. Furthermore, the World Health Organization suggests that it is important to inquire about women's sexual resumption and potential dyspareunia as part of a general well-being evaluation 2–6 weeks postpartum. However, in practice, healthcare providers frequently prioritize discussions about contraception over conversations about safe resumption of sexual intercourse, leaving many women without essential guidance. Despite the recognition of the importance of postpartum sexual health, existing guidelines and strategies often fall short of addressing this topic adequately (4, 12, 21).

The objective of this research is to investigate the factors contributing to early postpartum sexual intercourse among married women in Ethiopia, using data from the Performance Monitoring for Action (PMA) project. Unlike previous studies that used a closed population using logistic regression, this study employed a dynamic follow-up approach. Since participants were not all surveyed at the exact same time postpartum, the follow-up period varied, allowing this study to address this timing variation. Even though studies have examined postpartum intimate violence, they have not explored its relationship with early resumption of sexual intercourse.

## Methods

### Study area and period

PMA Ethiopia is a survey initiative to provide data on various reproductive, maternal, and newborn health (RMNH) indicators to support government decision-making at national and regional levels. The project has been conducting cross-sectional and cohort surveys to address a data gap, gathering data that is not currently captured by other major surveys, specifically focuses on assessing the extent of RMNH care services and the factors that hinder or help in providing such care, which was conducted at Amhara, Oromia, SNNP, and Addis Ababa (22).

PMA Ethiopia's Cohort 2, also known as the second cohort of PMA Ethiopia. The baseline interviews were held while they were pregnant or up to nine weeks postpartum, enumerators gathered data on the socio-demographic features of women, their pregnancy intentions, and the antenatal care services they had received. The data collection from baseline to 6-week follow-up survey was conducted between November 2021 and October 2022, while the one-year follow up was completed by August 2023. Women who met the requirements and agreed to participate were included in the cohort. A longitudinal dataset collected form eligible women at baseline, 6 weeks, 6 months, and one year after childbirth in selected areas (23–26). In this study, women who completed both the baseline and six-week follow-up assessments were included in the analysis.

### Study design

The study employed a cross-sectional design at baseline, Women eligible for the cohort and willing to participate in the follow-up were enrolled in the study.

## Population

### Source population

The source population comprised all women of reproductive age (15–49 years), either pregnant or those up to nine weeks postpartum at baseline in Ethiopia.

### Study population

The source population comprised all women of reproductive age (15–49 years), either pregnant or those up to nine weeks postpartum at baseline and willing to participate in the cohort in Ethiopia.

### Eligibility criteria

#### Inclusion criteria

The source population comprised all women of reproductive age (15–49 years), either pregnant or those up to nine weeks postpartum at baseline and willing to participate in the cohort during the study period in Ethiopia.

## Sample size determination

### Sample size determination PMA

PMA Ethiopia calculated the sample size by utilizing data from past PMA2020 surveys to predict the modern Contraceptive Prevalence Rate, address the design effect, and consider non-response. 217 enumeration areas (EAs) were chosen to achieve a 5% margin of error for modern Contraceptive Prevalence Rate estimates in each panel region, and PMA2021 updated to 162 EAs.

### The sample size included in this study

Out of the 10,389 women included in the baseline survey, 2,297 women (pregnant and those up to nine weeks postpartum) were followed up in the cohort study. The remaining 8,092 women were excluded from the cohort as they were neither pregnant nor postpartum. Of the 2,297 women, 1,796 were pregnant, 228 were 0–4 weeks postpartum, and 273 were 5–9 weeks postpartum. The 273 women in the 5–9 weeks postpartum group were asked about the six-week questionnaire at baseline, while the remaining 2,024 women (1,796 pregnant women and 228 women who were 0–4 weeks postpartum) were asked after six weeks. Of the 2,297 women, 2,144 were married, and 1,807 of them responded to the questionnaire on resuming sexual intercourse. The final sample size used for analysis was 1,807, of whom 512 were asked about resuming sex within six weeks postpartum.

### Sampling technique and procedure

PMA Ethiopia employed multistage stratified cluster sampling, where households were selected in sampled clusters or enumeration areas (EAs). EAs were selected with probability proportional to size within strata. In Amhara, Oromia, and SNNP regions, strata were created based on urban/rural residence. However, there is no strata applied for Addis Ababa. Within panel regions, a census of all households was conducted. From the census, enumerators identified all women who were aged 15–49 and regular members of the household. Women were screened and those who reported being pregnant or having given birth within nine weeks were eligible for the survey.

## Study variables

### Dependent variables

Early resumption of sexual intercourse (resume == 1, not resume = 0).

### Independent variables

#### Socio-demographic variables

Socio-demographic variables included in the analysis were Religion, Women age group, Partner age group, Strata, Education status of the women, Education status of the husband, Residence, Wealth quintile, Living together after marriage, Marriage history, Her husband has other wives, Intimate pressured sex, and Intimate forced sex.

#### Reproductive variables

Reproductive variables included in the analysis were Currently breastfeed, Current family planning user, CS delivery, Severe bleeding during delivery, Convulsion during delivery, High fever and foul-smelling discharge (<24 delivery), Retained placenta (<24 hr, >30 min), Desired pregnancy, Parity, and Delivery place.

### Operational definition

#### Follow-up period

The Cohort 2 PMA data study was conducted at baseline, 6 weeks, 6 months, and one year postpartum. For this study analysis, only data from the baseline and 6-week follow-up periods were used. Women who were pregnant, 0–4 weeks postpartum and 5–9 weeks postpartum were included in the cohort. Women in the 5–9 weeks postpartum group were administered the 6-week follow-up questionnaire at the same time as the baseline assessment while those who were pregnant or 0–4 weeks postpartum were asked the six-week postpartum assessment after 6 weeks from the baseline survey. Even though it is called six-week postpartum follow up, the six-week follow up assessment were administered between four week and almost ten weeks postpartum.

#### Intimate pressured sex

Intimate partner conflict for having sexual intercourse during pregnancy, without the use of physical force, but involving emotional or verbal pressure (23, 24).

#### Intimate forced sex

Intimate partner conflict while pregnant for having sexual intercourse using physical force (23, 24).

#### Survival time

Time from birth to six-week follow-up time (it is greater than six weeks for postpartum women during the baseline survey). It was calculated by subtracting the birth date from the interview date for six-month follow-up in the dataset. For the six-week follow-up, the minimum and maximum times to resume sexual intercourse were 30 and 68 days, respectively (23, 24).

#### Event

The resumption of sexual intercourse between childbirth and six-week postpartum follow-up period.

#### Early resumption of sexual intercourse

Sexual resumption occurred within six weeks postpartum (42 days) (4, 5, 27, 28).

### Data extraction tools and procedure

Survey was carried out in the initial interview to collect information on different socio-demographic aspects of women, those who were able and willing to give consent were enrolled in the follow-up survey. They were asked about sexual resumption and other reproductive health questions over the course of the year. Since this study objective is about early resumption of sexual intercourse, women who completed six-week follow-up assessment were included in the data extraction.

### Data quality management

To maintain data quality, the data was collected through training and ongoing monitoring for data collectors and supervisors during the data collection process. Daily checks of data were carried out, and field teams were given feedback to fix any discrepancies found. The study also incorporated data validation rules within the ODK system, minimizing entry errors and ensuring the reliability of the collected data.

### Data processing and analysis

Data were cleaned, processed, and analyzed using R statistical software. Multiple imputations were used for missing values of independent variables. Descriptive statistics were first conducted, followed by survival analysis using a stratified Cox proportional hazard model in R software version 4.4.1. Cox proportional hazard assumption was checked using Schoenfeld Residuals Plot and Schoenfeld Residuals test. Multi-collinearity was checked, women who had forced sex also had pressured sex from their partner, they were collinear to each other, and pressured sex was selected for multivariable cox model building even though the crude hazard ratio is significant for both variables. Backward variable selection method was used for variable selection. The model fitness was checked using martingale residual plot. The shared frailty model for the stratum was evaluated, but the frailty term was not significant, so cox proportional hazards model without frailty was used instead.

### Ethical approval

The data set was obtained from the PMA dataset website through email after announcing the overall objective of the study.

## Results

The participants included in the analysis were 512 within 42 days (six weeks) postpartum and 1,807 within 68 days postpartum.

### Socio-demographic variables

The majority of females were older than 25 years (66%), while 24% were aged 20–24, and 10% were between 15 and 19 years old. Partners aged between 15 and 19 years old were 1%, 9% were between 20 and 24 years old, and 90% were over the age of 25. Oromiya-2 had the highest percentage of participants at 27%, with SNNP-2 following at 18% and Amhara-2 at 16% in terms of regional representation.

When it comes to education, 44% of women had finished primary school, while 31% had never been to school. Likewise, primary education had been finished by 41% of husband, with 26% having no formal education. Most of the individuals (61%) resided in rural regions. According to wealth distribution data, 28% of the population were in the top quintile, while 19% were in the bottom and lower quintiles.

Regarding marriage, 61% of couples had lived together for more than five years, and 89% of women were married once. 92% of respondents said their husbands did not have other wives. 7% reported experience of pressured sex, and 5% reported experience of forced sex from their husband (Table 1).

**Table 1:** Socio-demographic variables.

Variable	Category	Asked about time resume within 42 days (6 weeks)	Asked about time resume within 72 days (10 weeks)
Religion	1. Orthodox	186 (0.36)	678 (0.38)
	2. Protestant	145 (0.28)	564 (0.31)
	3. Muslim	173 (0.34)	543 (0.30)
	4. Other	8 (0.02)	22 (0.01)
Women age group	15_19	50 (0.10)	175 (0.10)
	20–24	123 (0.24)	429 (0.24)
	>25	339 (0.66)	1203 (0.66)
Partner age group	15_19	5 (0.01)	28 (0.02)
	20–24	48 (0.09)	163 (0.09)
	>25	459 (0.90)	1616 (0.89)
Strata	10. Addis	41 (0.08)	225 (0.12)
	3. Amhara-1	39 (0.08)	127 (0.07)
	3. Amhara-2	84 (0.16)	266 (0.15)
	4. Oromiya-1	73 (0.14)	236 (0.13)
	4. Oromiya-2	137 (0.27)	434 (0.24)
	7. SNNP-1	45 (0.09)	158 (0.09)
	7. SNNP-2	93 (0.18)	361 (0.20)
Education status of the women	0. Never attended	157 (0.31)	489 (0.27)
	1. Primary	223 (0.44)	789 (0.44)
	2. Secondary	79 (0.15)	309 (0.17)
	3. Technical & vocational	23 (0.04)	76 (0.04)
	4. Higher	30 (0.06)	144 (0.08)
Education status of the husband	0. Never attended	134 (0.26)	407 (0.23)
	1. Primary	212 (0.41)	725 (0.40)
	2. Secondary	95 (0.19)	383 (0.21)
	3. Technical & vocational	19 (0.04)	74 (0.04)
	4. Higher	52 (0.10)	218 (0.12)
Residence	1. Urban	198 (0.39)	746 (0.41)
	2. Rural	314 (0.61)	1,061 (0.59)
wealth quintile	1. Lowest quintile	99 (0.19)	290 (0.16)
	2. Lower quintile	97 (0.19)	290 (0.16)
	3. Middle quintile	76 (0.15)	289 (0.16)
	4. Higher quintile	97 (0.19)	352 (0.19)
	5. Highest quintile	143 (0.28)	586 (0.32)
Living together after marriage	1_2	124 (0.24)	481 (0.27)
	3–5	76 (0.15)	258 (0.14)
	>5	312 (0.61)	1,068 (0.59)
Marriage history	1. Only once	458 (0.89)	1,625 (0.90)
	2. More than once	54 (0.11)	182 (0.10)
Her husband has other wives	0. No	472 (0.92)	1,680 (0.93)
	1. Yes	40 (0.08)	127 (0.07)
Intimate pressured sex during pregnancy	0. No	477 (0.93)	1,707 (0.94)
	1. Yes	35 (0.07)	100 (0.06)
Intimate forced sex during pregnancy	0. No	485(0.95)	1,718(0.95)
	1. Yes	27(0.05)	89(0.05)

Other - No religion non-believer, Traditional, Wakefeta, and Catholic.

### Maternal and reproductive health factors

Most women (85%) of the women gave birth vaginally, while 15% had cesarean sections. In terms of delivery, 14% faced severe bleeding, 11% suffered convulsions, and 12% had high fever or foul-smelling discharge shortly after giving birth. Furthermore, 4% of females indicated experiencing a retained placenta.

In terms of pregnancy desires, 68% of women wanted their pregnancy at the time, while 28% wanted it later, and 4% did not want it at all. Regarding to parity 59% of women had two or less parity, 18% had between three and four parity, and 23% had over 4 parity. Regarding to delivery, 38% of deliveries took place at government health centers, 34% occurred at home, and 25% were in government hospitals (Table 2).

**Table 2:** Maternal and reproductive health factors.

Variable	Category	Asked about time resume within 42 days (6weeks)	Asked about time resume within 72 days (10 weeks)
Currently breastfeed	0. No	8 (0.02)	21 (0.01)
	1. Yes	504 (0.98)	1,786 (0.99)
Current family planning user	0. No	496 (0.97)	1,582 (0.88)
	1. Yes	16 (0.03)	225 (0.12)
CS delivery	0. No	434 (0.85)	1,637 (0.91)
	1. Yes	78 (0.15)	170 (0.09)
Sever bleeding during Delivery	0. No	439 (0.86)	1,537 (0.85)
	1. Yes	73 (0.14)	270 (0.15)
Convulsion during delivery	0. No	455 (0.89)	1,608 (0.89)
	1. Yes	57 (0.11)	199 (0.11)
High fever and foul smelling discharge (<24 delivery)	0. No	453 (0.88)	1,584 (0.88)
	1. Yes	59 (0.12)	223 (0.12)
Retained placenta (<24 hr, >30 min)	0. No	490 (0.96)	1,700 (0.94)
	1. Yes	22 (0.04)	107 (0.06)
Desired pregnancy	1. Then	349 (0.68)	1,229 (0.68)
	2. Later	144 (0.28)	504 (0.28)
	3. Not at all	19 (0.04)	74 (0.04)
Parity	≤2	302 (0.59)	1129 (0.63)
	>4	92 (0.18)	292 (0.16)
	3–4	118 (0.23)	386 (0.21)
Delivery place	1. home	176 (0.34)	536 (0.30)
	11. Government hospital	126 (0.25)	500 (0.28)
	12. Government health center	195 (0.38)	701 (0.39)
	96. Other	15 (0.03)	70 (0.04)

Other - other private medical sector, NGO/Faith-based health facility, and Private hospital/clinic.

### Resume sex

**Figure 1:**
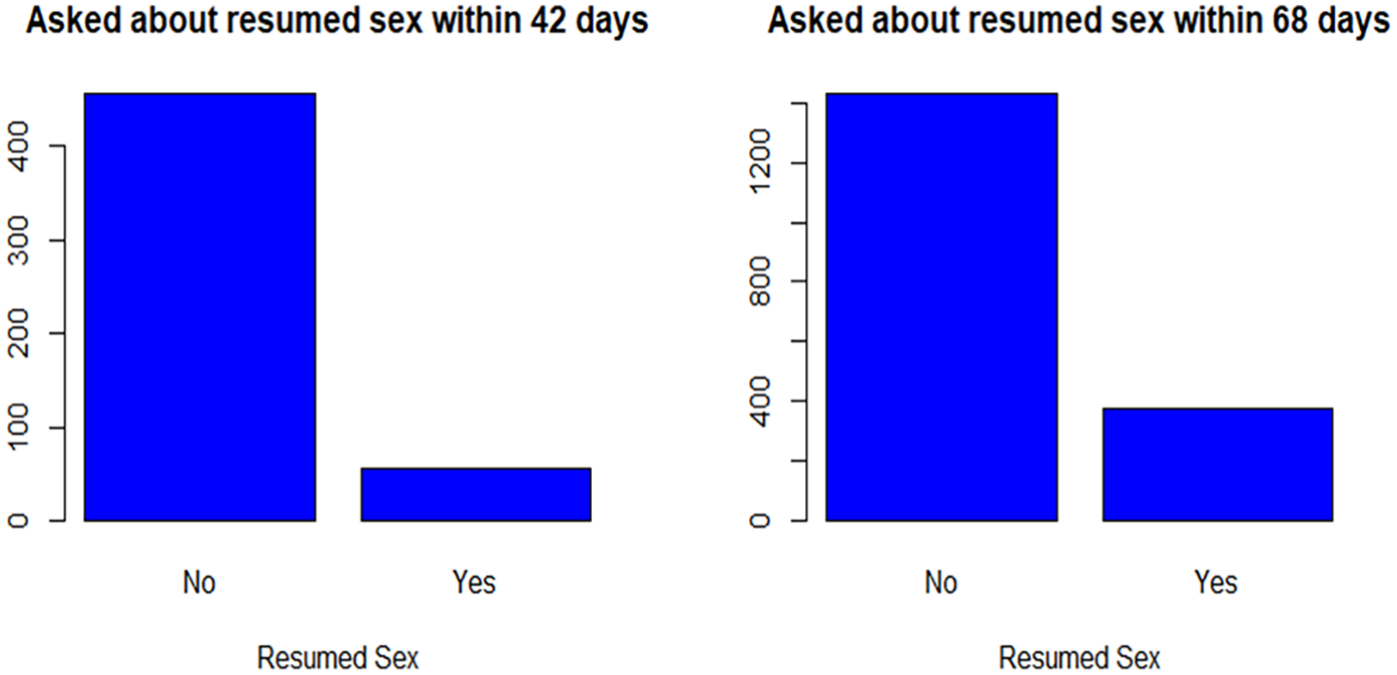
Resumed sex within six weeks and 68 days.

Out of the 512 participants, asked about resumed sex within 42 days postpartum (6 weeks), 56 (10.94%) had the event of interest (resumed sex). Among 1,807 participants, 379 (20.98%) had the event of interest by 68 days (9.7 weeks) (Figure 1).

### Survival probability

**Figure 2:**
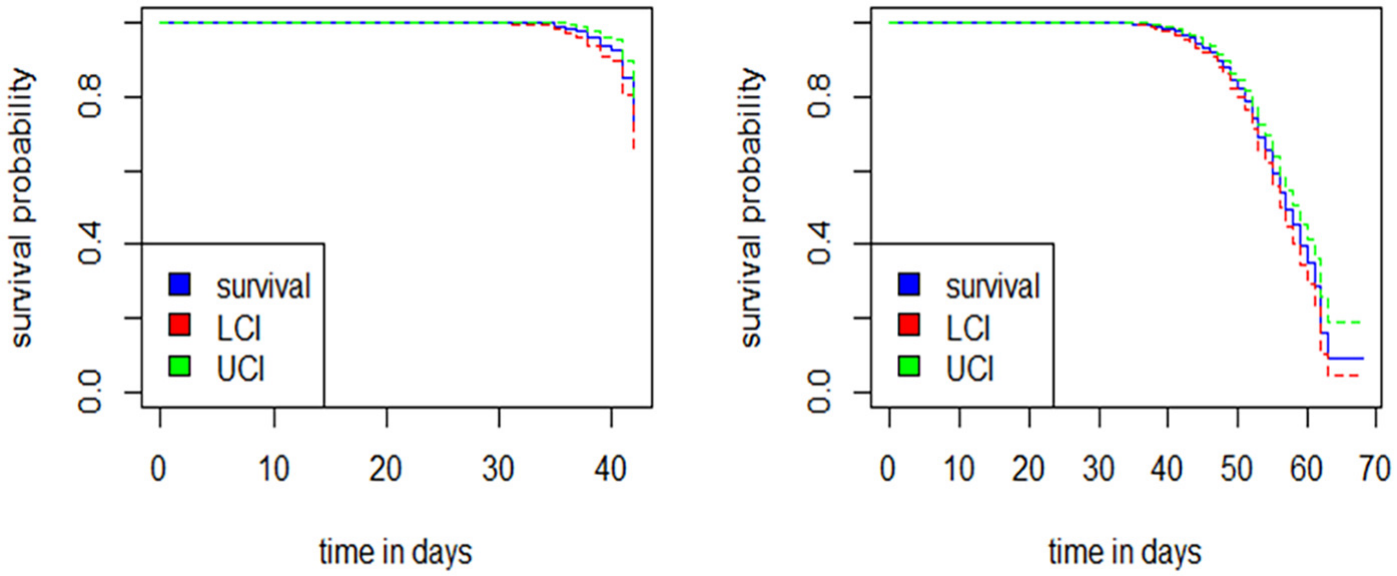
Survival probability of resumed sex within six weeks and 68 days.

The median survival time was 57 days. The survival probabilities at 42 and 68 days were 0.71 and 0.09 respectively, with an incidence rate of 45 per 10,000 person-days. The restricted mean survival time was 56.7 days (95% CI: 56–59) (Figure 2).

**Figure 3:**
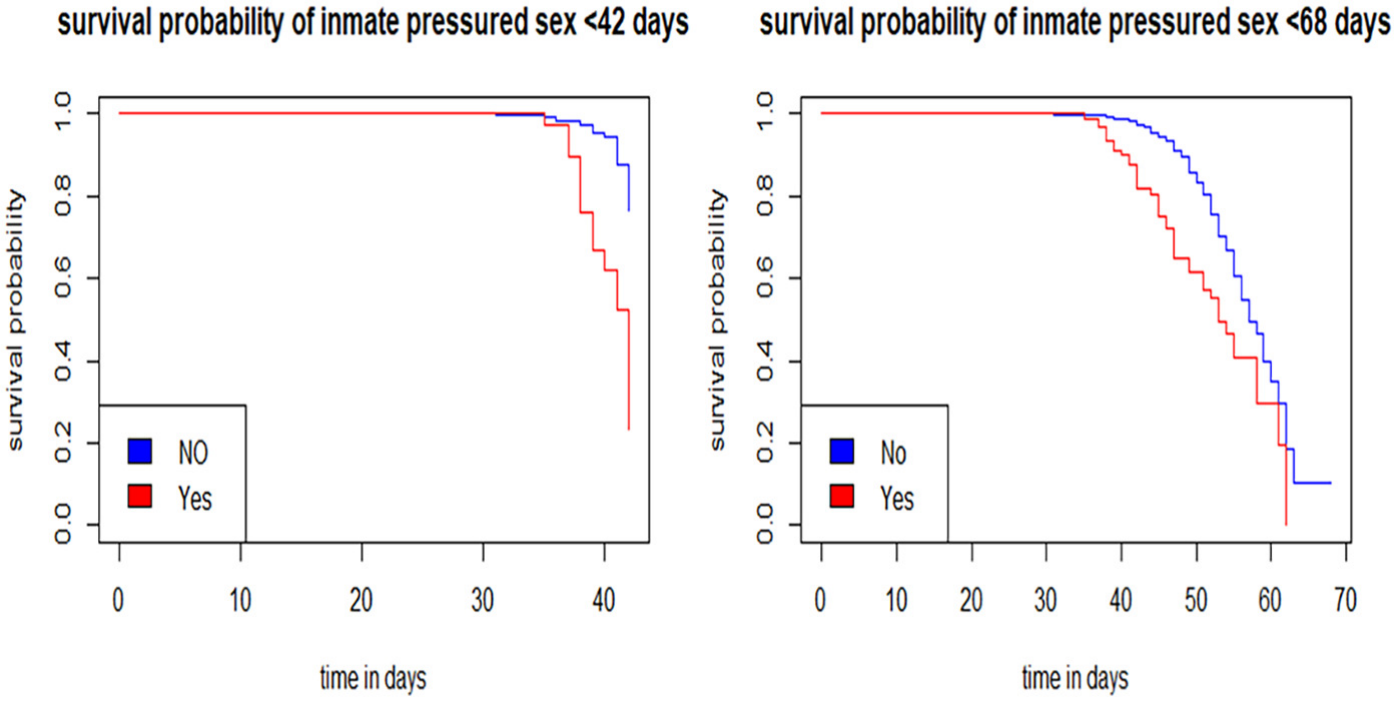
Survival probability of resumed sex for intimate pressured sex.

Individuals who reported experiencing intimate partner-pressured sex had lower survival probabilities compared to those who did not. Over time, the difference in survival probabilities between these two groups remained relatively small (Figure 3).

### Proportional hazards assumption test

The solid line of smoothed Schoenfeld residuals is mostly flat with minor fluctuations, indicating that the proportional hazards (PH) assumption holds. The dashed confidence bands contain the solid line, further supporting this assumption. The scattered points are randomly distributed, showing no clear trend, which also suggests that the PH assumption is not violated. The Schoenfeld residual test results indicate that the proportional hazards assumption holds for all variables (all *p* > 0.05. The global test (*p* = 0.25) supports the overall assumption (Supplementary file).

### Multivariable Cox proportional hazards model (early sexual resumption)

The hazard of early sexual resumption among women who felt pressured by their partners was 5.56 times more likely to resume sexual intercourse compared to those who did not experience any pressure (Table 3).

**Table 3:** Cox proportional hazards model for resumed sex within six weeks.

Variable	Category	Resumed sex		CHR (95% CI)	AHR (95% CI)
		Yes	No		
Intimate partner-pressured sex	0. No	40 (7.81)	437 (85.35)	Ref.	
	1. Yes	16 (3.12)	19 (3.71)	5.56 (3.11, 9.94)***	5.99 (3.27, 10.98)***
Cesarean section delivery	0. No	48 (9.38)	386 (75.39)	Ref.	
	1. Yes	8 (1.56)	70 (13.67)	0.91 (0.43, 1.93)	0.50 (0.10, 2.59)

AHR, adjusted hazard ratio; CHR, crude hazard ratio; CI, confidence interval; Ref-reference category, ***> - <0.001.

### Multivariable Cox proportional hazards model (up to nearly 10 weeks-68 days)

The hazard of resuming sexual intercourse among women who felt pressured by their partners was 2.13 times more likely to resume sexual intercourse compared to those who did not experience any pressure (Table 4).

**Table 4:** Cox proportional hazards model for resumed sex within 68 days.

Variable	Category	Resumed sex		CHR (95%CI)	AHR (95%CI)
		Yes	No		
Intimate partner-pressured sex	0. No	335 (18.54)	1,372 (75.93)	Ref.	
	1. Yes	44 (2.43)	56 (3.1)	2.13 (1.56, 2.92)***	2.14 (1.47, 3.12)***
Cesarean section delivery	0. No	350 (19.37)	1,285 (71.11)	Ref.	
	1. Yes	29 (1.6)	143 (7.91)	0.62 (0.42, 0.91)	0.72 (0.42, 1.22)
Retained placenta after delivery	0. No	354 (19.59)	1,346 (74.49)	Ref.	
	1. Yes	25 (1.38)	82 (4.54)	0.82 (0.55, 1.23)	0.77 (0.52, 1.14)
Desired pregnancy	1. Then	262 (14.5)	967 (53.51)	Ref.	
	2. Later	103 (5.7)	401 (22.19)	0.91 (0.72, 1.14)	0.88 (0.70, 1.11)
	3. Not at all	14 (0.77)	60 (3.32)	0.76 (0.44, 1.29)	0.78(0.46, 1.33)

AHR, adjusted hazard ratio; CHR, crude hazard ratio; CI, confidence interval; Ref-reference category, *** - <0.001.

### Model fitness

**Figure 4:**
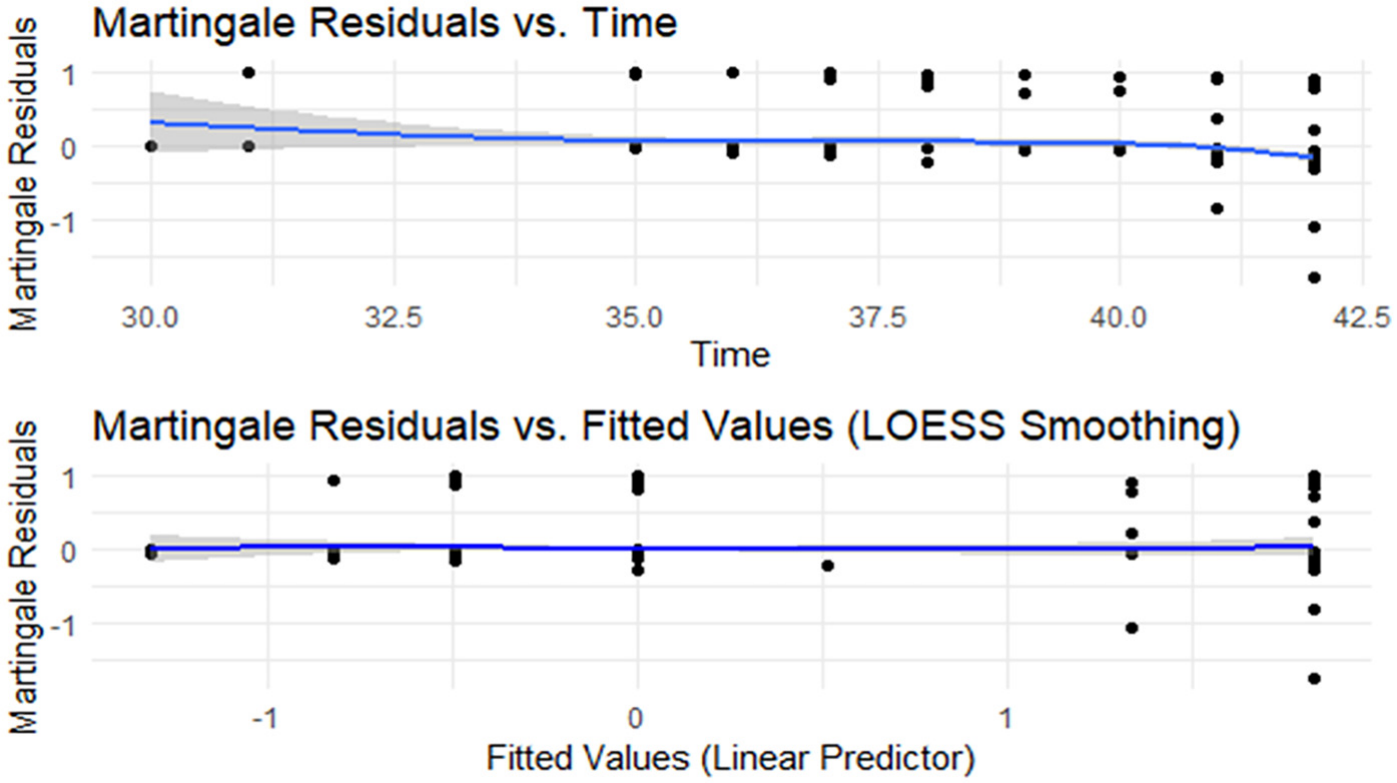
Model fitness using martingale residuals.

The martingale residual plot suggests that the model's fit was reasonable (Figure 4).

## Discussion

The study revealed that 29% of participants resumed sexual intercourse before the recommended 42 days postpartum (survival probability 0.71), and 91% (survival probability 9%) had resumed sex by 68 days (9.7 weeks). The finding of this research was supported by studies done in Ethiopia and Nigeria (4, 14, 16). Although the puerperal period is meant for physical and psychological recovery early resumption of sexual intercourse may be driven by cultural, societal, and familial pressures that force women to engage in sexual intercourse earlier than recommended (1, 13, 14, 29, 30). Additionally, in Ethiopia, limited access to comprehensive postpartum care often leaves women without sufficient information about the risks associated with early sexual intercourse. Cultural norms also play a significant role, as discussing sexual matters openly is considered disrespectful, resulting in health professionals frequently avoiding these important conversations (31).

The finding of this study was lower than in Sub-Saharan Africa, Uganda, and Nigeria (5, 12, 32). This discrepancy may be attributed to religious and cultural practices in Ethiopia, where sexual intercourse before the baptism of an infant is often discouraged. Additionally, the dynamic nature of this follow-up study could contribute to the differences, as participants were not surveyed at the same time postpartum, leading to variations in their responses. Another factor that may account for these differences is the variation in the periods when the studies were conducted. Additionally, this study finding are lower than studies done in Ethiopia (6, 18, 33). The discrepancy may be attributed to differences in methodology and time, as well as Ethiopia's multicultural nature. Studies conducted in specific areas may yield different results compared to the nationwide data used in this study.

The finding of this study was higher than studies done in Uganda, and Brazil (27, 34). The possible reason may be due to Cultural influence or social pressures that may lead to an earlier resumption of sexual intercourse postpartum, especially related to maintaining marital harmony or fulfilling partner expectations. The finding of this study was higher than a study previously conducted in Ethiopia (11). This discrepancy may be because the current study uses nationwide data, whereas the previous study focused on a specific area of Ethiopia.

The median time in this study was 57 days which is consistent with the previous study done in Nigeria (56 days/8 weeks) (1). Half of the women resumed sexual intercourse after 8 weeks postpartum, which is in line with the recommendation to wait at least 6 weeks. The timing depends on both physiological and psychological readiness, as well as open communication with their partner regarding sexual matters (21, 28, 35).

The finding that 91% of individuals resumed sexual intercourse by 68 days (9 weeks and 5 days) post-delivery may be due to recovery in both physical and emotional well-being after 9 weeks compared to before 6 weeks postpartum. This high rate after 9 weeks compared to before 6 weeks postpartum suggests the challenges of postpartum recovery, which needs time include physical healing, hormonal changes, and adjustments later than sooner. It may be due to factors such as effective communication with partners, emotional support, and proper guidance from healthcare providers regarding sexual health are crucial in this transition (1, 13, 14, 29, 30).

The finding that early resumption of sex was 1.97 per 100 person-weeks at 6 weeks and 3.1 per 100 person-weeks at 68 days postpartum highlights the gradual increase in sexual intercourse as parents recover physically and emotionally as well as emphasizes the frequency with which postpartum women in this study resumed sexual intercourse early. This trend difference may be due to physical recovery and emotional readiness over time, which can lead to the consensual resumption of sexual intercourse (1, 13, 14, 29, 30, 36).

This study's findings on intimate partner pressure reveal an alarming trend, with women who felt pressured by their partners being 5.56 times more likely to resume sexual intercourse early. This reflects a broader issue of gender inequality and power dynamics within intimate relationships. Studies in Ethiopia and other sub-Saharan African contexts consistently reveal that male partners frequently dominate household choices, particularly in matters concerning sexual relations. This power disparity can result in women feeling pressured to comply with their partner's sexual requests, regardless of their readiness, either physically or emotionally (36, 37). In male-dominated communities like Ethiopia, women often have limited control over their sexual health. During the postpartum period, women often feel unable to decline sexual advances from their partners, leading to high rates of intimate partner violence and sexual coercion. Pressure like this can result in the early resumption of sexual intercourse (7–10, 38).

Women who felt pressured by their partners had a significantly higher likelihood of engaging in sexual intercourse within 42 days after giving birth [AHR: 5.99 (95% CI: 3.27–10.98)] and within 68 days postpartum [AHR: 2.14 (95% CI: 1.47–3.12)]. Even though the strength of association was lower, intimate partner violence was a predictor of resumption of sexual intercourse even after 6 weeks post-delivery (7–10, 38, 39).

## Conclusion

This study showed high prevalence of early resumption of sexual intercourse among postpartum women in Ethiopia. Half of the participants resumed sex at 8 weeks postpartum and most of them at ten weeks postpartum. Intimate partner pressure was a significant predictor of early resumption of sexual intercourse.

## Data Availability

The data analyzed in this study is subject to the following licenses/restrictions: It was accessed after a clear description of the study objective to PMA data owners. They offer the data only to eligible persons. We will send the data if it is needed during the review process. Requests to access these datasets should be directed to https://www.pmadata.org/countries/ethiopia.
