# Supplementary material for: Intimate partner sexual violence and early resumption of sexual intercourse among married postpartum women in Ethiopia: a survival analysis using Performance Monitoring for Action data
Source: Front Glob Womens Health. 2025 Apr 30;6:1499316. doi: 10.3389/fgwh.2025.1499316 (PMC12075142; doi:10.3389/fgwh.2025.1499316)
Supplement: Supplementary file 1 [file Table1.docx]

The solid line of smoothed Schoenfeld residuals is mostly flat with minor fluctuations, indicating that the proportional hazards (PH) assumption holds. The dashed confidence bands contain the solid line, further supporting this assumption. The scattered points are randomly distributed, showing no clear trend, which also suggests that the PH assumption is not violated (Supportive Figure 1).


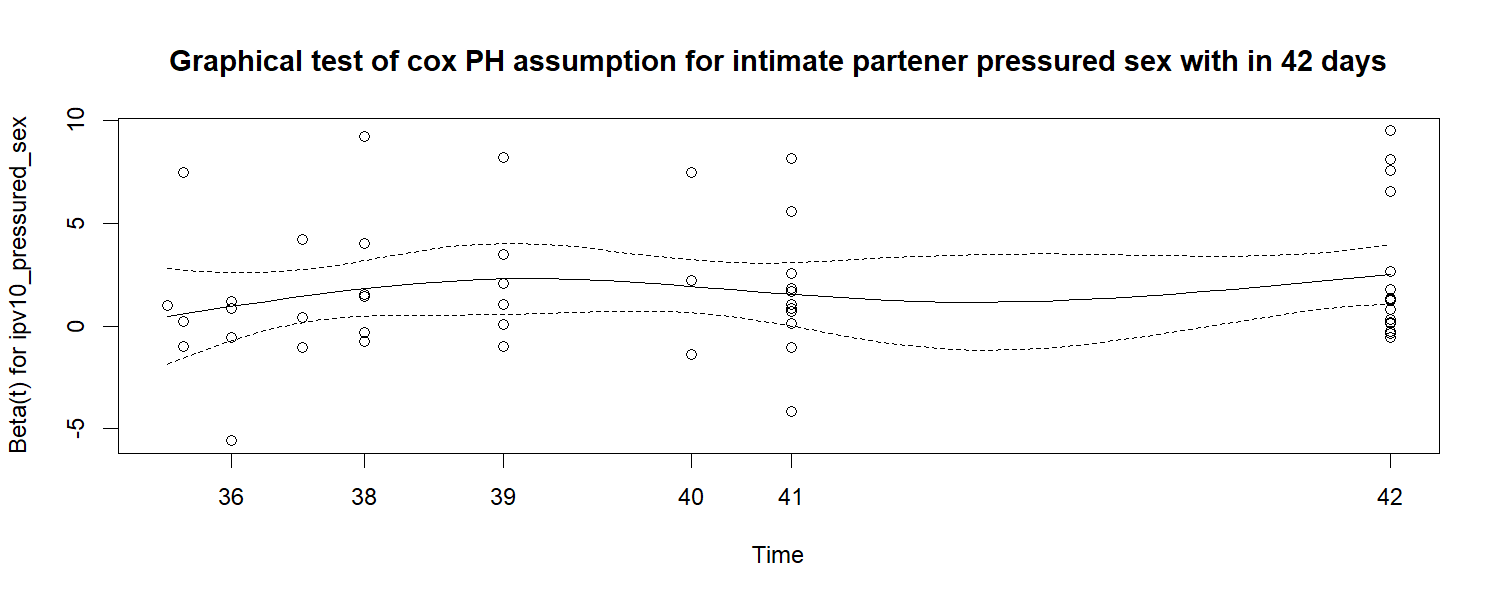


Supportive Figure 1 Schoenfeld residual plot of resumption of sexual intercourse within 42 days

The Schoenfeld residual test results indicate that the proportional hazards assumption holds for all variables (p>0.05). The global test (p = 0.25) suggests no overall violation of the proportional hazards’ assumption, indicating that the model is appropriate for Cox regression (Supportive Table 1).

Supportive Table 1 Schoenfeld residual test of resumption of sexual intercourse within 42 days

| Variable | chisq | df | p |
| --- | --- | --- | --- |
| Parity | 1.42 | 2 | 0.49 |
| Religion | 2.53 | 2 | 0.28 |
| Women age group | 4.88 | 2 | 0.09 |
| Her husband has other wives | 2.7 | 1 | 0.1 |
| wealth quintile | 1.78 | 4 | 0.78 |
| Residence | 0.22 | 1 | 0.64 |
| Desired pregnancy | 0.93 | 2 | 0.63 |
| Living together after marriage | 1.41 | 2 | 0.5 |
| Education status of the women | 3.36 | 4 | 0.5 |
| Education status of the husband | 1.47 | 4 | 0.83 |
| Marriage history | 0.41 | 1 | 0.52 |
| Currently breastfeed | 1.76 | 1 | 0.18 |
| Delivery place | 7.67 | 3 | 0.053 |
| CS delivery | 0.02 | 1 | 0.9 |
| Convulsion during delivery | 0.26 | 1 | 0.61 |
| Sever bleeding during  Delivery | 0.02 | 1 | 0.88 |
| Retained placenta (<24hr, >30 minute) | 2.14 | 1 | 0.14 |
| High fever and foul  smelling discharge (<24 delivery) | 0.37 | 1 | 0.54 |
| Intimate pressured sex during pregnancy | 0.32 | 1 | 0.57 |
| Current family planning user | 1.53 | 1 | 0.22 |
| GLOBAL | 41.42 | 36 | 0.25 |
